# Are We in a De‐Globalization Process? The Evidence from Global Trade During 2007–2017

**DOI:** 10.1002/gch2.202000096

**Published:** 2021-05-27

**Authors:** Xiaomeng Li, Chen Shen, Hongbo Cai, Qinghua Chen

**Affiliations:** ^1^ School of Systems Science Beijing Normal University Beijing 100875 China; ^2^ New England Complex Systems Institute Cambridge MA 02139 USA; ^3^ Business School Beijing Normal University Beijing China

**Keywords:** de‐globalization, expectation maximization, international trade, trade purity indicator, trade resistance

## Abstract

Through the analysis of statistical data, some scholars believe that the globalization of trade is declining, and de‐globalization has become a trend, even since the financial crisis in 2008. However, the superficial decline of global trade volume cannot be taken as a corollary of the de‐globalization. It needs go deep into the structural analysis, and “globalization or de‐globalization” should be discussed by analyzing whether the global trade structure has changed. This paper finds that during 2007–2017, the global trade resistances are clearly classified, and trade resistance in the global community has increased significantly. Second, an expectation maximization (EM) algorithm is applied to divide global trade relations into two categories: intimate trade relations, whose barriers are mainly related to geographical distance; and unfriendly trade relations with high artificial barriers. Third, the trade purity indicator (TPI) is introduced to describe the trade environment of countries, and its evolution indicates after the financial crisis and for quite a long time, the structure of global trade has not changed much. And it shows some deterioration trend and structural adjustment after 2015, which indicates an opportunity for the emergence of de‐globalization in such an international environment full of uncertainty and challenges.

## Graphical abstract

The development of international trade has met great challenges, and some scholars even declare that de‐globalization started after the 2008 economic crisis. It is proposed that the trade purity indicator (TPI) can reveal the structural change rather than the apparent change of global trade volume. Here the TPI analysis shows different conclusions.

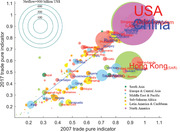

## 1 Introduction

International trade benefits regional development and makes Pareto improvement according to economic theories of free markets and comparative advantages.^[^ ^1^, ^2^ ^]^ Since the Second World War, global trade has shown a great development. In particular, from 1950 to 2008, the total value of global trade exports increased from 62 billion US dollars to 16 trillion US dollars. For more than half a century, not only the developed countries in Europe and America, but also many developing countries are actively participating in the wave of globalization.^[^ ^3^, ^4^ ^]^ And the formation of the global industrial chain has a great impact on the economy of each country.^[^ ^5^, ^6^ ^]^ However, trade brings benefits to economic development but trade disputes frequently emerge. Some countries accuse trade partners of setting inappropriate tariff barriers, which are unfair and harmful to the profits of domestic producers. After the financial crisis in 2008, the scale and growth of international trade have shrunk significantly, and the growth rate of trade has been less than 3% for four consecutive years. Such a low growth rate has been rare in the past 50 years.

Regarding the merchandise movement, after a sharp decline in 2008, the general expectation was that trade would continue to grow at rates similar to those previous to the crisis. But this is not the case. Trade volume grew by an average of 3.5% from 2009 to 2018, which is much slower than the 7.6% average growth before the 2008 financial crisis.^[^ ^7^ ^]^ Meanwhile, some countries set up investment restrictions and trade barriers. Recently, there has been a more severe test. Like they say, it never rains but it pours. The coronavirus disease pandemic has been pervasive since the beginning of 2020, shrinking the global economy due to reduced movements of goods and services.^[^ ^8^ ^]^ We are now at a zero growth rate in trade, which is understandable on the back of the Sino‐US trade war and several other protectionist waves, such as the Brexit. Due to these circumstances, there is a high risk of the world economy to move into a depression, and the growing evidences suggests that we may live in a period of de‐globalization that began a decade ago.^[^ ^9^, ^10^, ^11^ ^]^

At present, the economics have different dependence on international trade, and the adjustment of global industrial chain will have a great impact on some countries.^[^ ^12^, ^13^, ^14^ ^]^ An insight into the trend of globalization or de‐globalization can help them optimize decision‐making and avoid economic stagnation. So “has globalization slowed down or ended and de‐globalization emerged”? These issues need to be clarified eagerly.^[^ ^15^ ^]^ In existing literature, some studies measure globalization or de‐globalization mainly through directly comparing the increase of exports to the increase of GDP.^[^ ^16^ ^]^ These studies are based on the analysis on the phenomenon or statistics data, which are still simplified local analyses. Later, some scholars measured the degree of de‐globalizaion based on entropy theory,^[^ ^17^ ^]^ or used the reciprocal of trade liberalization index.^[^ ^18^ ^]^ And some literature quantified the trade restrictiveness indices (including anti‐dumping duties) or analyzed the structural characteristics of global trade network.^[^ ^19^, ^20^ ^]^ However, globalization or de‐globalization should be discussed from the structure of global trade, which needs an overall perspective and structural index analysis. But these literature are still limited to certain countries and lack global vision. And the studies based on network theory always lost important information when extracting backbone network, and the analysis is not comprehensive and scientific. So whether de‐globalization has emerged, or when will it occur? We need to explore the structural changes of global trade pattern, and build some systematic and core indicators that are not easily disturbed by local disturbance in data.

This paper quantifies the multilateral trade resistance and defines TPI which can objectively reflect the trade environment of countries/regions and measure the trend of globalization or de‐globalization.^[^ ^11^ ^]^ It is of strategic significance for the countries with strong dependence on foreign trade to grasp the evolution trend of world trade environment in time. Firstly, this paper gives quantitative description of changes in trade resistance between countries during 2007–2017. Here, it proposes a new paradigm to quantify the trade resistance and shows that global trade relations can be separated into two distinct categories, where the first is controlled by geographical distance, and the second is controlled by other aspects of trade barriers. Then, by distinguishing the two categories, it contributes to an understanding of different factors affecting trade and defines a country's TPI as the expected probability to have a pure trade partner. It shows the evidence of an alienating trend of global trade relations, and indicates that we are possibly in de‐globalization process since 2015, based on the significant changes of global trade structure. Here the interpretation of trade pattern changes can help different countries/regions make rational and scientific response to the global challenges.

## 2 Distribution and Dynamics of Trade Resistance

### 2.1 Application and Extending of Gravity Model

As one of the most successful models in trade research, a generalization of Newton's universal gravity model in Physics was pioneeringly applied to trade by Isard and Tinbergen.^[^ ^21^, ^22^ ^]^ In the generalized gravity model, the trade volume *F*_*i*, *j*_ between countries *i* and *j*,

(1) $$F_{i,j}\propto \frac{{\left(m_{i}\cdot m_{j}\right)}^{\alpha }}{{\left(d_{i,j}\right)}^{\beta }}$$

is determined by three factors: the economy sizes *m*_*i*_ and *m*_*j*_, usually measured by GDP;^[^ ^23^ ^]^ and their distance, *d*_*i*, *j*_, usually measured by minimum geographic distance or trade transport distance.^[^ ^24^, ^25^ ^]^ Here, α and β are parameters of the scaling with economy size and distance.

The effectiveness of the gravity model in explaining trade flows inspired vast amounts of theoretical and empirical literature with good performance in modeling trade flows,^[^ ^26^, ^27^ ^]^ where the variation in the flows could be explained by the fitted relationship.^[^ ^28^ ^]^ In order to further enhance the fitting, more factors were introduced,^[^ ^29^, ^30^ ^]^ such as tariffs,^[^ ^31^ ^]^ policy barriers,^[^ ^32^ ^]^ communication costs, information costs, enforcement costs, exchange rates, and legal or regulatory factors.^[^ ^9^, ^33^ ^]^

Scholars have been concerned that the incessant addition of factors would inevitably succumb to subjectivity, inconsistency, inexhaustibility, and the inaccessibility of reliable data. Accordingly, they introduced composite indicators into the model. The structural gravity model is a typical example,^[^ ^28^ ^]^

(2) $$F_{i,j}=\frac{m_{i}\cdot m_{j}}{\sum_{i}m_{i}}{\left(\frac{t_{i,j}}{H_{i}\cdot P_{j}}\right)}^{1-\sigma }$$

In this model, two kinds of trade resistance are considered: *H*_*i*_ and *P*_*j*_ are determined by the countries individually, and all the relations between the two countries are represented as a composite indicator *t*_*i*, *j*_. This innovation leads to a concise model and avoids the tendency to exhaustively identifying explanatory variables as in previous literature. The idea of composite indicators was also mentioned in the Ricardian model^[^ ^34^ ^]^ and heterogeneous firms models.^[^ ^35^, ^36^ ^]^ However, the authors did not quantify these resistances directly but through setting formulas for specific factors in empirical analysis.^[^ ^31^ ^]^

Different from the existing research, the trade resistance (TR) model in this paper uses the optimization method to quantify the multilateral trade resistance that best matches the world trade flow data,

(3) $$F_{i,j}\propto \frac{{\left(m_{i}\cdot m_{j}\right)}^{\alpha }}{r_{i,j}}$$

where *r*_*i*, *j*_ is the multilateral trade resistance between country *i* and *j*, which could be seen as a combination of *t*_*i*, *j*_ and *H*_*i*_ · *P*_*j*_ in structural gravity model, but is more concise and suitable for quantitative analysis with the econometric model as,

(4) $$\ln F_{i,j}=c+\alpha \ln\left(m_{i}\cdot m_{j}\right)-\ln r_{i,j}+\varepsilon_{i,j}$$

And *r*_*i*, *j*_ could be obtained by ordinary least squares (OLS). In addition, there are zero values in bilateral migration data, which is also a problem that has long puzzled researchers.^[^ ^37^, ^38^, ^39^ ^]^ Here, we use the pseudo maximum likelihood (PML) method to preprocess the zero value flow; for details, please find the details in Supporting Information S4.

### 2.2 Distribution of Trade Resistance

**Figure 1:**
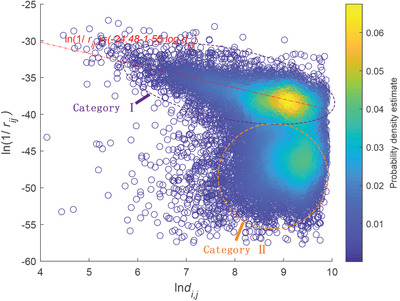
Relationship of trade resistance and geographic distance between countries/regions in 2017. In category I, ln (1/*r*_*i*, *j*_) decreases as the logarithm of geographic distance increases in an approximate linear fashion, as ln (1/*r*_*i*, *j*_) = −24.48 − 1.55ln *d*_*i*, *j*_. But there is no correlation between resistance and geographic distance in category II. Due to the size and shape of the earth, the maximum distance between two countries is about 20 000 (≈*e* ^10^) km.

Take the year of 2017 as the example, the empirical distribution of ln *r* *_i_*, *_j_* is clearly classified.^[^ ^11^ ^]^ **Figure** **1** shows the relation between trade resistance and geographic distance described as ln (1/*r* *_i_*, *_j_*) and ln (*d* *_i_*, *_j_*). The circles represent all trade resistances between 198 countries/regions (for each pair of countries (*i*, *j*), whether they have empirical trade flow data or not). The larger the reciprocal value, the smaller the trade resistance and the easier the trade flow. It more intuitively shows the density of the reciprocals of the trade resistance at different geographic distances with different colors, and two parts are clearly differentiated. The upper part (category I) has a significant downward shift with geographic distance increasing, which can be better described by the equation ln (1/*r* *_i, j_*) = −24.48 − 1.55ln *d* *_i, j_*. The average ln (1/*r* *_i, j_*) in the lower part (category II) is independent of geographic distance. It is consistent with the dichotomy of trade frictions as “natural” and “unnatural.”^[^ ^25^ ^]^ Geographic distance is an example of “natural” trade friction, and “unnatural” trade resistances include tariffs and other “artificial” or “policy” barriers.^[^ ^11^, ^31^, ^40^ ^]^ This classified characteristic exists extensively throughout the whole period of 2007–2017.

**Figure 2:**
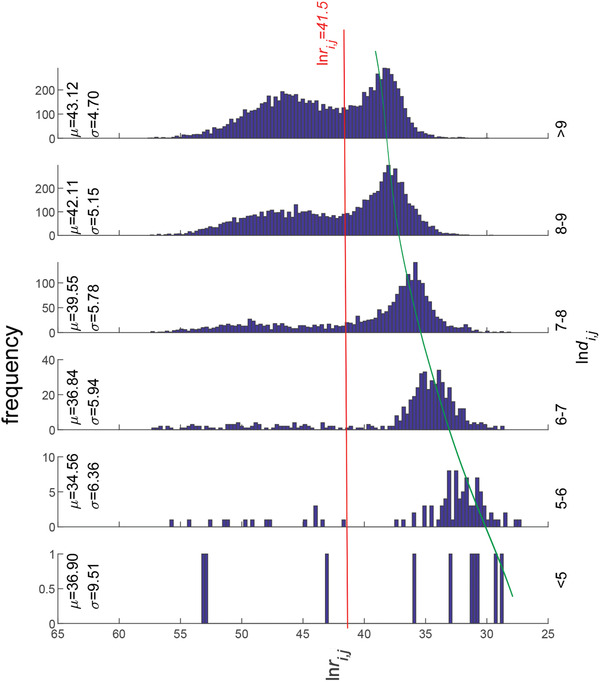
Distribution of trade resistance in 2017. The logarithm of geographic distance is divided into six intervals as: <5, 5–6, 6–7, 7–8, 8–9, >9. Below the reference line (red dashed line), the trade resistance increases as the geographic distance gets bigger. This tendency is shown by the green line.

**Figure** **2** shows histograms of trade resistance for different distance ranges. We see that the trade for countries in the larger trade resistance group is independent of distance while the average value of the trade for countries in the low trade resistance group increases significantly with the growth of geographic distance (as the green line shows). Furthermore, the standard deviation of trade resistance decreases as geographic distance becomes larger, which implies tendency to converge.

**Figure 3:**
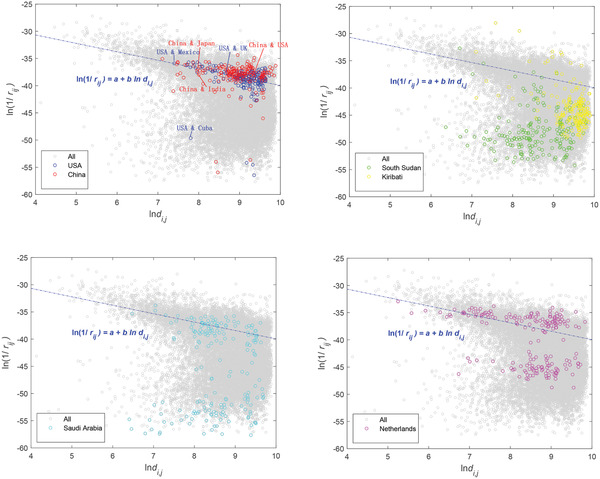
Trade resistance for select countries in 2017. For the United States and China, most of their trade relations with other countries in the world are good (in category I), with only a few exceptions. On the contrary, there are high trade resistances between South Sudan/Kiribati and other countries. Saudi Arabia and the Netherlands are somewhere in between.

**Figure** **3**a shows the trade resistances between all the countries/regions and China (red circles); between all the countries/regions and the United States (blue circles). As a typical trade surplus economy, China's resistances mainly belong to category I. The trade resistances between China and most other trade partners (e.g., United States, Japan, and India) can be approximated by their geographic distance respectively. The United States is a similar case. As the largest importer, the United States’ trade resistances are mainly determined by natural factors, and belong to category I (with exceptions like that with Cuba). Figure 3b shows some typical countries that have high trade barriers with many other countries, such as South Sudan and Kiribati. For these countries, most of the trade relationships belong to category II with high “artificial barriers.” Saudi Arabia (Figure 3c) and the Netherlands (Figure 3d) represent another type of case. The trade relations of Saudi Arabia (or the Netherlands) and the world could be roughly equally divided into two groups, with one located in category I, and the other in category II.

In addition, we compare the fitting effects of *F*_*i*, *j*_, using geographic distance *d*_*i*, *j*_ (Equation (1)) and trade resistance *r*_*ij*_ (Equation (3)). Unsurprisingly, it can fit the trade flows with significantly smaller sum of square error (*SSE*) than geographic distance since the new model has more degrees of freedom (*DF*) with more parameters. However, the adjusted coefficient (Adj. *R* ^2^) of determination has also been improved (see **Table** **1**).

**Table 1:** Comparison of fitting effect between models with geographical distance or trade resistance

	Model	$\hat{\alpha }$	$\hat{\beta }$	*DF*	*SSE*	*Adj*. *R^2^*
1	$F_{i,j}\propto \frac{{\left(m_{i}\cdot m_{j}\right)}^{\alpha }}{d_{i,j}^{\beta }}$	1.702[1.685,1.720]	1.469[1.398,1.540]	2	9.900*E*5	0.543
2	$F_{i,j}\propto \frac{{\left(m_{i}\cdot m_{j}\right)}^{\alpha }}{r_{i,j}}$	1.066[0.816,1.377]	—	19504	2.096*E*5	0.806

### 2.3 Dynamics of Trade Resistance

**Figure 4:**
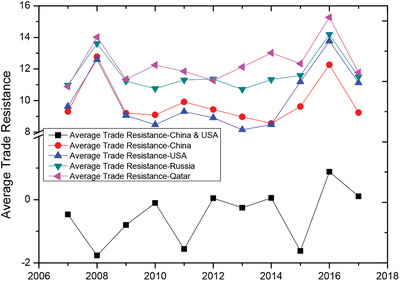
Development of trade resistance in the decade 2007–2017. The average trade resistance for representative countries increased.

We find the high correlations between the trade resistance change of the first peak and quantified transport costs by United Nations Conference on Trade and Development (UNCTAD),^[^ ^41^ ^]^ with Pearson corr.=0.06 and sig.=0.87. This supports our speculation that the first category's resistance is mainly determined by geographic distance. Here we try to fix the first peak, mainly focusing on the evolution of the second peak. In the past decade, the second peak has a right‐shift trend, indicating that the artificial barriers increased greatly regardless of overall cost changes. **Figure** **4** exhibits the dynamics of average trade resistances for some representative countries with the rest of the world. Here we use the difference of average trade resistance and the first peak. A positive value indicates that the resistance is higher than the peak value of the category I; and negative one is lower than the peak value.

Here compared with Russia and Qatar (green triangle and pink triangle), China and the United States have lower trade resistance with the rest of the world (red circle and blue triangle); and the resistance between China and the United States (black square) is comparatively low, even smaller than the peak value of category I. It means China and the United States have very close trade relations, but during this decade, their trade resistance has increased. In the decade from 2007 to 2017, the growth of trade resistance between China and United States (3.4 times), is much higher than that between them and the rest of the world (around 1 time for China and 1.2 times for the United States). This increase in trade resistance has a huge impact on Sino‐US trade relations, and has also attracted the attention of stakeholders of participating countries.

## 3 Country's Trade Pure Indicator

### 3.1 Expectation Maximization Algorithm

For each pair of countries *i* and *j*, the trade resistance *r*_*i*, *j*_ is quantified with the TR model and we find that trade resistances are always classifying distributed and can be separated into two groups. As shown above, the data $R=\{\ln r_{1,2},\ldots ,\ln r_{i,j},\ldots \}$ can be divided into two categories: I is mainly related to natural factors such as geographic distance *d*_*i*, *j*_; and II is affected by more artificial barriers than natural factors.

(5) $$\ln r_{i,j}=\{\begin{array}{ll} a+b\ln d_{i,j}+\eta_{i,j} & (r_{i,j})\in I\\ \xi_{i,j} & (r_{i,j})\in \text{II} \end{array}$$

Here *a*, *b* are constants. η_*i*, *j*_ and ξ_*i*, *j*_ are normal distribution random variables with different means and standard deviations, η_*i*, *j*_ ≈ *N*(0, σ_1_) and ξ_*i*, *j*_ ≈ *N*(μ, σ_2_). How to estimate parameters $\Theta =\{\mu ,\sigma_{1},\sigma_{2},a,b\}$ based observed data R and put each ln *r*_*i*, *j*_ into appropriate category?

In order to solve the parameter problem of two mixed distributions, we apply the expectation maximization (EM) algorithm.^[^ ^42^ ^]^ In statistics, the EM algorithm is an iterative method to find the maximum likelihood or maximum a posteriori (MAP) estimates of the parameters in statistical models, where the algorithm depends on unobserved latent variables.^[^ ^43^, ^44^, ^45^ ^]^ The workflow of the EM algorithm and the details could be found in Figure S1, Supporting Information. We have the good fitting results and the estimated parameters show relatively stable characteristics during 2007–2017 (Table S2, Supporting Information). Here we use the Kolmogorov–Smirnov test to return the decision for the null hypothesis that the data of ln *r*_*i*, *j*_ comes from a classified distribution with the parameter estimation $\hat{\Theta }$. The fitting results and quantified ln *r*_*ij*_ are subject to the same distribution and consistent with theoretical predictions for each year in the period of 2007–2017 at a significance level of α = 0.1. It confirms the previous hypothesis that trade relations can be divided into two categories. It can also be seen that the mean value of the second peak $\hat{\mu }$ is increasing, the standard deviation ${\hat{\sigma }}_{2}$ is also increasing, and the distribution becomes more scattered.

### 3.2 Trade Purity Indicator

τ_*i*, *j*_ in Equation (S2), Supporting Information, can describe the probability of ln *r*_*i*, *j*_ belonging to category I and it has great significance in describing trade relations between countries. For each country *i*, we define the TPI *I*_*i*_ as the expectation of the probability that its trade resistance belongs to the first category,

(6) $$I_{i}=\left\langle {\hat{\tau }}_{i,j}\right\rangle =\frac{\sum_{j\neq i}{\hat{\tau }}_{i,j}}{n-1}$$

*I*_*i*_ reflects the mean of good trade relationship; a value above 0.5 means that the relationship between a country and its trading partners is mainly in the first category. The higher the value of *I*_*i*_, the better the trade relationship a country has with the world; the lower the value, the worse the relationship. We will use this index to discuss the global trade situation and its dynamics in the next section.

## 4 Alienation of Global Trade Relations

### 4.1 Global Trend of Trade Environment

TPI is based on the quantification of multilateral trade resistance. According to the analysis on the distribution characteristics of trade resistance, TPI can quantitatively describe the trade environment of each country, region or the whole world. The calculation process of TPI verifies that trade resistances can be divided into two categories, one of which is related to natural factors, mainly geographical distance. In fact, the traditional gravity model takes geographical distance as the only factor of trade barrier, which is not rigorous. In this paper, trade relations are divided into two categories. One is only hindered by geographical distance, with no obvious artificial obstacle, and it is consistent with the successful application of gravity model in trade research field. Besides, this paper also points out the shortcomings of the gravity model, and here, TR model can also be used for quantitative analysis of trade relations with higher artificial barriers.

**Figure 5:**
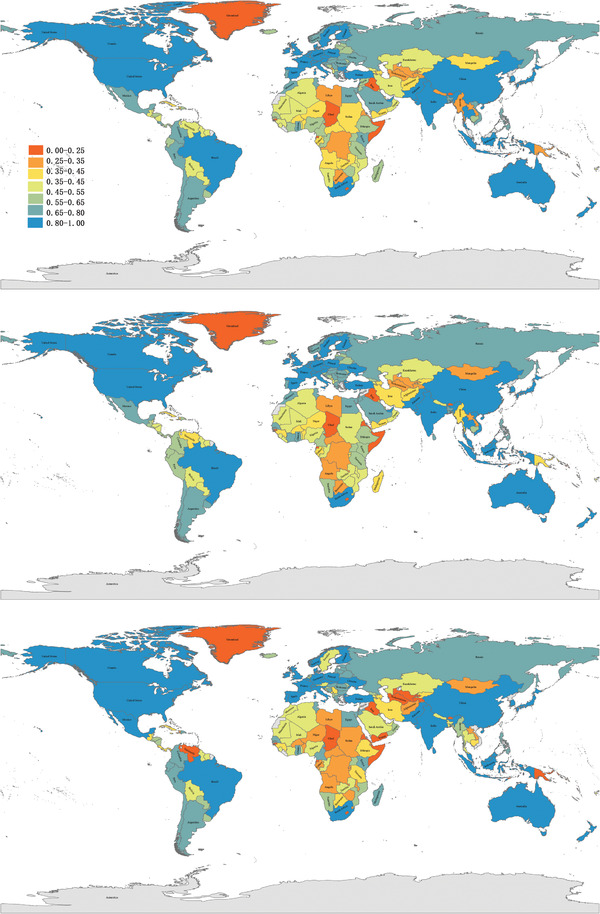
Trade purity indicator *I* for countries/regions in a) 2007, b) 2012, c) 2017. Different colors denote different TPI values. The higher the value, the bluer the color; and the lower, the redder.

**Figures** **5**a–c exhibit the TPI values for all countries/regions in 2007, 2012, and 2017, respectively. Here, the countries or regions with blue color have large *I*_*i*_, meaning that more of their trade relations belong to category I, such as the United States, countries of European Union, China, Japan, South Korea, Malaysia, Singapore, South Africa, and Australia. For these countries, most trade resistances are regulated by natural factors such as geographic distance and have no obvious trade barriers. In contrast, red and orange indicate countries with high trade barriers, such as a group of African and West Asian countries.

**Figure 6:**
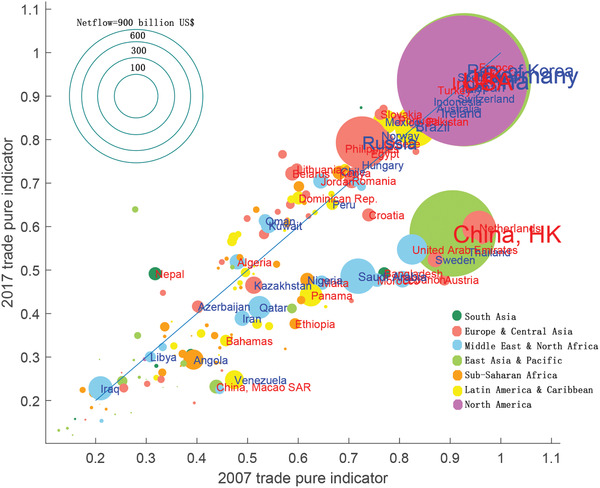
Change of trade purity indicator *I* from 2007 to 2017. The countries/regions below the diagonal line (slope=1) have a reduction in TPI.

**Figure** **6** shows the global change of TPI in the last decade. Each circle represents a country/region. The size of the circle represents the trade volume in 2017, and the color indicates the region in which the country is located. Red label means that the country/region had a trade deficit in 2017, and blue ones show a trade surplus. Generally, countries/regions with large trade volumes tend to have a higher TPI, where trade resistance is a negative facilitator. The countries below the blue diagonal have declined in TPI in the period. In contrast, the countries above the diagonal have improved TPI and decreased the artificial barriers with some countries in recent years.

During 2007–2017, generally speaking, the number of countries/regions with deteriorating trade environment is more than that with improving ones. And some countries/regions, such as Hong Kong (CHN), Netherlands, Ethiopia, Saudi Arabia, and the United Arab Emirates, have obviously decreased TPIs. For other countries, such as Russia, Mexico, Philippines, and Nepal, their TPIs have improved in recent years, which means these economies reduced the trade barriers. We discuss some representative countries/regions in the following section.

### 4.2 Change of Trade Structure and Emergence of De‐globalization

**Figure 7:**
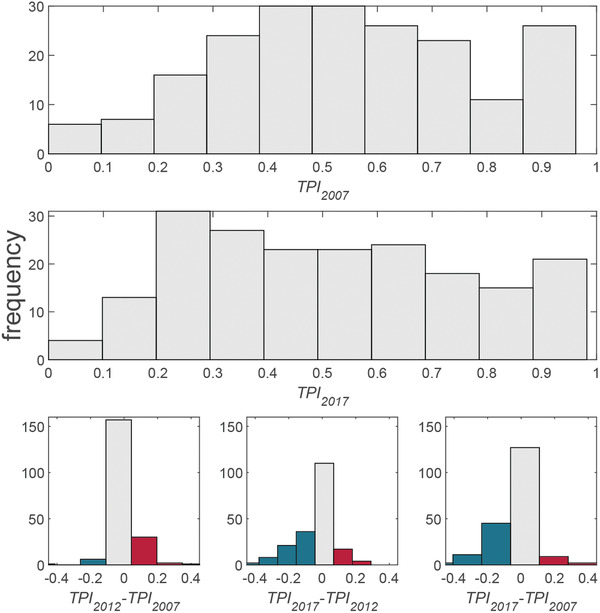
The distribution of TPI and ΔTPI. The distribution of TPI in 2017 has a significant left shift (becoming smaller) compared with 2007. This decrease of TPI is mainly in the period of 2012–2017.

It analyzes the dynamics of the TPI over the past decade, and the change of TPI can also clearly reveal the evolution of trade relations in various countries. **Figure** **7** shows the TPI change in distribution. It indicates that the distribution of TPI in 2017 has a significant left shift (becoming smaller) compared with 2007, where more TPIs have decreased and been found in the negative (reduced) area (subplot in bottom right). For the period 2007–2012, the change of distribution is more symmetrical (subplot in bottom left). But the Δ TPI from 2012 to 2017 moves to the left, which shows that the decrease of TPI is mainly in the period of 2012–2017. The Gini coefficient of the distribution is 0.23 in 2007 and 0.32 in 2017, which indicates the gap in trade relations between countries is increasing. The global mean of TPI decreases from 0.55 in 2007 to 0.52 in 2017, which indicates the continuous increasing of average trade resistances globally and the global downward trend of trade purity indicator.

**Figure 8:**
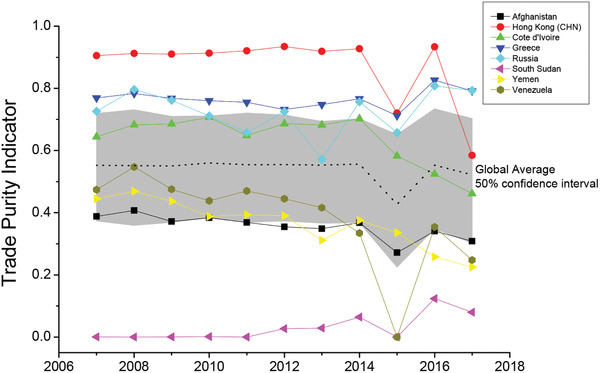
Evolution of trade purity indicator. Some representative cases. In the past decade, TPI of many countries/regions has been declining, including the ones with a good trade environment, such as Hong Kong (CHN) and Cote d'Ivoire; and the ones with poor infrastructure, like Afghanistan, Yemen and Venezuela. Although a few countries have improved TPI, such as Russia, Greece, and South Sudan, on the whole, global trade relations were indeed alienating during 2007–2017.

But, can the decline of TPI indicate that we have entered the stage of de‐globalization? This is not enough and it is necessary to further analyze whether the distribution characteristics of TPI and global trade structure have changed. Here, several representative countries/regions show different trends of TPI (**Figure** **8**) as follows.

The black dotted line represents the average TPI, and the grey shadow area contains the countries/regions whose TPI is 50% of the middle. First, from Figure 8, TPI of several representative countries/regions declined as a whole in 2015, representing the overall increase of unnatural trade barriers and the deterioration of trade environment. This phenomenon is not difficult to explain because there is a significant decline in the global trade volume in 2009 and 2015, including exports and imports. However, in 2009, it was caused by the financial crisis, and the GDP of many countries/regions also declined. Therefore, TPI excluded the influence of economic size, and measured the trade environment more objectively. It believed that the trade environment in 2009 did not change greatly, but the decline of trade volume is caused by the reduction of production. However, in 2015, the steady growth of GDP and the significant reduction of trade volume make the quantified TPI decrease significantly, which objectively reflects the change of trade resistance.

Despite the particularity of 2015, we can still see from Figure 8 that before 2015, although the TPI was decreasing, it was generally stable. But after 2015, TPI has a relatively obvious decline, which also corresponds to the change of trade structure of some countries or regions. Hong Kong (CHN) is a typical case. As one of the most important international ports, Hong Kong maintained good trade relations with most countries for a long time. And in 2017, Hong Kong's net trade flow was still the third largest in the world, over 394 billion US$, but its TPI showed an obvious downward trend.

**Figure 9:**
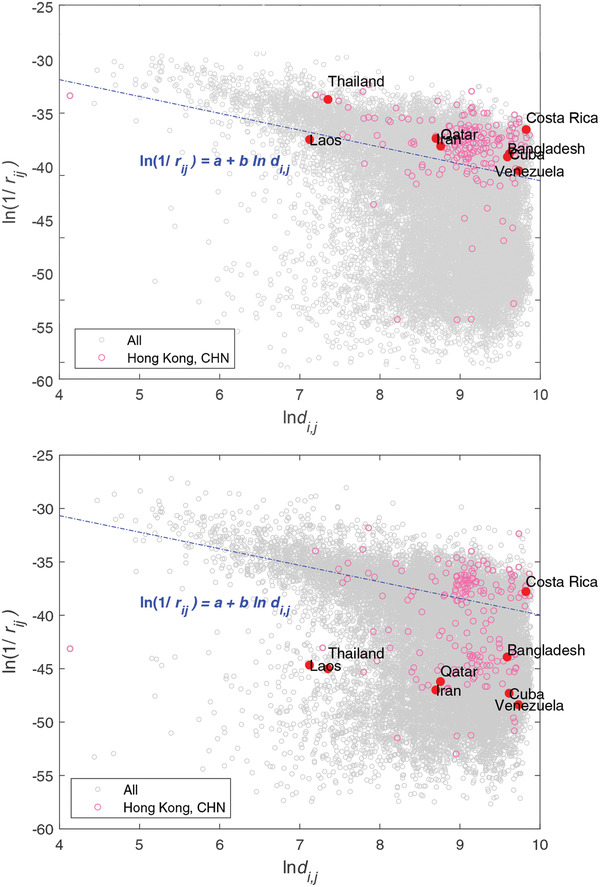
Trade purity indicator of Hong Kong (CHN) in 2016 (above) and 2017 (below). Some countries have increased trade resistances with Hong Kong in the period from 2016 to 2017.

So the traditional statistical methods will not find significant changes in Hong Kong's trade environment and the trend of de‐globalization. However, Hong Kong's trade purity indicator fell by 22.3% and 37.4% in 2015 and 2017, respectively (compared with the value of the previous year). In recent years, Hong Kong's GDP has been growing steadily and the trade volume kept increasing in 2017, but the variance of trade volume between Hong Kong and different countries also increased by 19.2%. The countries that increase their resistance with Hong Kong include: Qatar, Iran, Cuba, Venezuela, Thailand, Bangladesh, and Laos. **Figure** **9** shows the change of trade relationships between Hong Kong and other countries from 2016 to 2017. Obviously, the trade structure of Hong Kong has changed a lot, which could be detected by the change of TPI.

### 4.3 Typical Factors Affecting Trade Environment

This paper attempts to discuss the common factors that affect the trade environment, through the analysis of some representative countries or regions.

First, natural disasters, queasy political situation and civil war will seriously affect a country's trade environment. In the UN list of least developed countries, Afghanistan, Yemen, and South Sudan are the least developed countries in the world, and civil wars have had a great impact on their economic development and trade environment. During 2007–2017, for Afghanistan and Yemen, their trade purity indicators are similar, and both show a downward trend, with a decline of 20.5% and 49.4%, respectively (Figure 8). For South Sudan, it had barely traded with any other countries before its independence in July 2011. Since then, the trade environment of South Sudan has been improving,^[^ ^46^ ^]^ with TPI increases since 2012, and now its closest trade partners include Uganda, Kenya, Ukraine, China, and South Africa.

Second, accession to the multilateral free trade agreement (such as World Trade Organization and WTO) will help to improve the trade environment. Russia had an improving trade environment in recent years, despite a slight decline in Russia's trade purity indicator during 2010–2014. Russia joined the World Trade Organization (WTO) in 2012, and it obviously improved Russia's trading environment later by lowering the import tariffs.^[^ ^47^ ^]^ Figure 8 shows that Russia's TPI has increased significantly since 2014.

In addition, a sudden economic or political crisis will affect the trade environment, and this impact may last for a long time. Greece lies at the southern end of the Balkan Peninsula in southeastern Europe, and it is a developed country and a member of the European Union (EU) and North Atlantic Treaty Organization (NATO). Thus, Greece has a good trading environment and a higher TPI. In 2009, Greece was deeply in a debt crisis, which brought serious economic problems (including trade problems). Generally, in Greece, during recent decades, some structural reforms and adjustments have been implemented, more or less successfully.^[^ ^48^ ^]^ From 2010 to 2014, Greece's trade purity indicator declined accordingly, but it began to rise from 2015 and eventually recovered and surpassed the level of 2007.

Otherwise, be hostile to other countries especially to the influential powers, will worsen the trade environment within a certain range. As a founding member of OPEC, Venezuela is a typical mineral country,^[^ ^49^, ^50^ ^]^ with oil making up one‐third of its entire GDP.^[^ ^50^, ^51^ ^]^ Throughout most of the 20th century, Venezuela maintained friendly relations with most Latin American and Western nations. But the relation between Venezuela and the United States deteriorated from 2002.^[^ ^52^ ^]^ Venezuela is looking to improve trade relations with the Latin American zone, the EU and China; however, these years, Venezuela's trade purity indicator continues to decline.

## 5 Conclusion and Discussion

After the 2008 financial crisis, the global trade growth slows down compared with the increase of world GDP. Meanwhile, the trade disputes frequently emerge and some scholars believe that the trend of de‐globalization has started. But whether it has the transition from globalization to de‐globalization needs to be deduced from the analysis on global trade structure. It is not only a political and economic issue, but also scientific one.

First, this paper proposes a new framework for global trade structure research based on the quantitative and evolutionary analysis on the multilateral trade resistances between countries/regions. It uses a new paradigm and establish the TR model. And this method could avoid the shortcomings in the practice of introducing more influencing factors as the existing literature did.

Then, with the data from the UN Comtrade Database, we quantified the trade resistance of 198 countries/regions. It shows that the quantified trade resistance clearly obeys a classified distribution for each year during 2007–2017. This implies that the trade relationships of 198 countries can be divided into two categories. Based on the analysis on the distribution characteristics of trade resistances, we define and indicate the TPI for countries as the share of category I relations in all. On this basis, the study not only gives us a clearer understanding of the current trade pattern, but also shows us the changes of the global trade situation.

The analysis of TPI evolution shows the alienation of global trade relations in recent decade. It certifies that after the 2008 financial crisis and for quite a long time, the structure of global trade has not changed much. And it shows some deterioration trend and structural adjustment of great impact since 2015, which is precisely an opportunity for the emergency of de‐globalization. Unlike some existing literature believes that de‐globalization has emerged since the 2008, which is just based on analysis on the changes in statistical data, the fact that global trade barriers are increasing and global trade structure is changing deserves the attention of researchers and politicians.

There have been some discordant arguments in global trade recently; for example, some governments have accused other countries of setting unfair tariff. Sino‐US trade friction is a typical case under the influence of this trend of thought, which could affect development of regional economies.^[^ ^53^, ^54^, ^55^ ^]^ The sudden change of TPI in 2015 indicates that the global trade structure has indeed changed, which means that the process of de‐globalization may have begun. However, whether it really enters into the process of de‐globalization still needs to be analyzed with more empirical data in the later stage, which will provide the basis for policy regulation of some countries. This paper could become evidence for the intensity of current trade disputes, and the challenge is finding a solution to further deterioration.

In theory, our research is not a completely independent innovation but based on previous models and ideas, such as Anderson's structural gravity model. In that theory, three integration indexes *t*_*i*, *j*_, *P*_*j*_, and *H*_*i*_ are proposed. However, the indexes are coupled, which is not distinguishable, and the authors were forced to give some specific equations for these indexes when estimating. Here we directly use the empirical data for regression and get the estimation of trade resistance. The discovery of the classified distribution gives us a good chance to analyze the international trade purity of each country.

Besides, there are still some problems that need to be solved, including the asymmetry of actual trade flows, what factors determine resistance and how to decide them, and the way to help a policy maker improve global trade in a manner that is benign to a wide range of countries. These topics can be studied under the theoretical framework of this paper, which are also the focus of our future research.

## Conflict of Interest

The authors declare no conflict of interest.

## Supporting information

Supporting Information

## Data Availability

All data, models, or code generated or used during the study are available from the corresponding author by request.
