# Supplementary material for: Are We in a De‐Globalization Process? The Evidence from Global Trade During 2007–2017
Source: Glob Chall. 2021 May 27;5(8):2000096. doi: 10.1002/gch2.202000096 (PMC8335849; doi:10.1002/gch2.202000096)
Supplement: Supplementary file 1 — Supporting Information [file GCH2-5-2000096-s001.pdf]

© 2021 Wiley-VCH GmbH

Global Challenges
Open Access

Supporting Information

for *Global Challenges,* DOI: 10.1002/gch2.202000096

Are We in a De-Globalization Process? The Evidence from Global Trade During 2007–2017

*Xiaomeng Li, Chen Shen, Hongbo Cai, and Qinghua Chen**

Supplementary Material

Xiaomeng Li[1], Chen Shen[2], Hongbo Cai[4], and Qinghua Chen[1,2,3]

[1] School of Systems Science, Beijing Normal University - Beijing, China
[2] New England Complex Systems Institute - Cambridge, MA, USA
[3] Department of Chemistry, Brandeis University - Waltham, MA, USA
[4] Business School, Beijing Normal University, Beijing, China

S1 Data Source

In this paper, we use trade data from the UN Comtrade Database, including 198 countries or regions. We measure the total of trade by the aggregated Goods data in the Harmonized Commodity Description and Coding Systems (HS) commodities. When the flow data reported by the importers and exporters are not identical, we use the importers' report if available.

For GDP, we use the World Bank National Accounts Data and OECD National Accounts Data. It is calculated without making deductions for depreciation of fabricated assets or for depletion and degradation of natural resources. Data are in current U.S. dollars.

There are several methods for calculating geographic distance. Considering that some countries have multiple import and export ports, we use the central coordinates to calculate geographical distance. A full description of the data sources is provided in Table S1.

Table S1: Data Description

Indicator	Indicator Description	Data Source
Trade Flows	Country-to-Country Trade Volume (in current US$), from UN Comtrade Database, for "Goods" and "HS" commodities, annual data from 2007-2017.	`https://comtrade.un.org/data/`
GDP	GDP (in current US$) for countries, annual data from 2007-2017, from World Bank database.	`https://data.worldbank.org/indicator/NY.GDP.MKTP.CD`
Distance	Distance between geographic centre of countries (in km). Calculated from the central coordinate (centroids) data of countries or regions from the worldmap group of Harvard University.	`https://worldmap.harvard.edu/data/geonode:country_centroids_az8`

S2 Details of Expectation Maximization (EM) Algorithm

The EM algorithm seeks to obtain the MLE (maximum likelihood estimation) of the marginal likelihood by iteratively applying the expectation step (E step)

and maximization likelihood step (M step), with $t = 1, 2, ...$ representing the iteration times. The detail process of EM algorithm is as follows:

- **Expectation step (E step):** In step t, based on the last estimation of the parameters $\hat{\Theta}^{(t-1)}$, calculate the expected value of probability of belonging to a certain category.
  Calculate separately the probabilities for data $\ln r_{i,j}$ belonging to category I and category II.

$$\begin{aligned} p_1(r_{i,j} \mid \hat{\Theta}^{(t-1)}) &= \frac{1}{\sqrt{2\pi}\sigma_1} \exp \frac{-[\ln r_{i,j} - (a + b \ln d_{i,j})]^2}{2\sigma_1^2}, \\ p_2(r_{i,j} \mid \hat{\Theta}^{(t-1)}) &= \frac{1}{\sqrt{2\pi}\sigma_2} \exp \frac{-[\ln r_{i,j} - \mu]^2}{2\sigma_2^2}. \end{aligned} \tag{1}$$

  Then normalize them as

$$\hat{\tau}_{i,j}^{(t)} = \frac{p_1(r_{i,j} \mid \hat{\Theta}^{(t-1)})}{p_1(r_{i,j} \mid \hat{\Theta}^{(t-1)}) + p_2(r_{i,j} \mid \hat{\Theta}^{(t-1)})}. \tag{2}$$

  The unobserved latent variables $\Theta_\tau = \{\tau_{1,2}, \tau_{1,3}, ..., \tau_{i,j}, ...\}$, where $\tau_{i,j}$ $(0 \leq \tau_{i,j} \leq 1)$ represents the probability for trade resistance $\ln r_{i,j}$ belonging to category I.
- **Maximization likelihood step (M step):** Based on the $\hat{\Theta}_\tau^{(t)}$ obtained from E step, we find the parameters estimation $\Theta^{(t)}$ that maximizes this likelihood.
  The likelihood function L of occurring $\boldsymbol{R}$ is multiplying the expectation probability of all trade resistances. The optimum value of $\Theta^{(t)}$ based giving $\boldsymbol{R}$ and $\hat{\Theta}_\tau^{(t)}$ could be calculated from that function

$$\begin{aligned} \hat{\Theta}^{(t)} &= \max_{\Theta} \log L(\boldsymbol{R}; \Theta \mid \hat{\Theta}_\tau^{(t)}) \\ &= \max_{\Theta} \sum_{i \neq j} \log\{\hat{\tau}_{i,j}^{(t)} \cdot p_1(r_{i,j} \mid \Theta) + (1 - \hat{\tau}_{i,j}^{(t)}) \cdot p_2(r_{i,j} \mid \Theta)\}. \end{aligned} \tag{3}$$

From the random initial value of $\hat{\Theta}^{(0)}$, we repeat the iterative process of E and M steps until the likelihood function converges and the parameters $\hat{\Theta}$ are stable. In this paper, we run the algorithm for 50 iterations.

$$L(\boldsymbol{R}; \Theta, \Theta_\tau) = \prod_{i \neq j} \{\underbrace{\tau_{i,j} \cdot p_1(r_{i,j} \mid \Theta)}_{Category \quad I} + \underbrace{(1 - \tau_{i,j}) \cdot p_2(r_{i,j} \mid \Theta)}_{Category \quad II}\}.$$

S3 Fitting Result of EM Algorithm

Figure S1 shows the workflow of EM algorithm. For the year of 2007, 2012 and 2017, fitting results by EM algorithm are in Figure S1 (b)-(d), where the gray

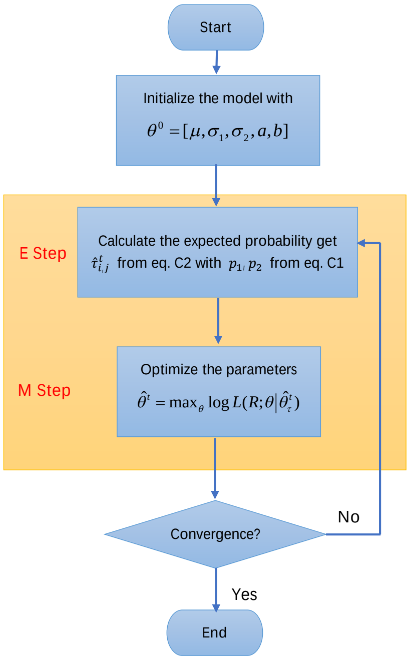

(a) workflow of EM algorithm

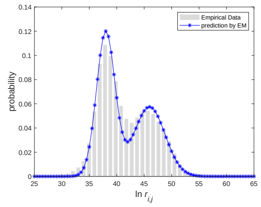

(b) Fitting for Data in 2012

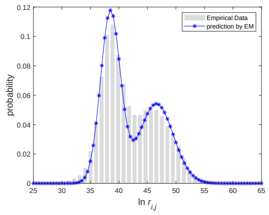

(c) Fitting for Data in 2007

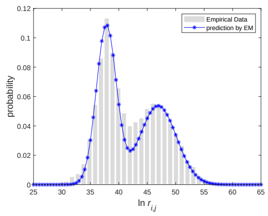

(d) Fitting for Data in in 2017

Fig. S1: Workflow Chart of EM Algorithm and the Fitting Results. (a) Workflow of EM algorithm; (b)-(d) are fitting results by EM. The empirical trade resistance distributions are consistent with theoretical predictions, demonstrated by good fitting.

columns are the distribution of the logarithm of trade resistance $\ln r_{ij}$ and the blue stars show the theoretical prediction of equation 3 of the main text. And we have the good fitting results by EM algorithm and the estimated parameters show relatively stable characteristics during 2007-2017 (Table S2). Here we use the Kolmogorov-Smirnov test to return the decision for the null hypothesis that the data of $\ln r_{i,j}$ comes from a classified distribution with the parameter estimation $\hat{\Theta}$. p is the p-value of the KS test. The fitting results and quantified $\ln r_{ij}$ are subject to the same distribution and consistent with theoretical predictions for each year in the period of 2007-2017 at a significance level of $\alpha = 0.1$. It confirms the previous hypothesis that trade relations can be divided into two categories.

Table S2: Estimations of parameters

	$\hat{a}$	$\hat{b}$	$\hat{\mu}$	$\hat{\delta_1}$	$\hat{\delta_2}$	KS statistics
2007	25.50	1.45	45.97	1.68	2.82	0.035 (p=0.29)
2008	28.34	1.27	48.77	2.03	2.81	0.023 (p=0.78)
2009	22.73	1.42	43.13	1.72	2.83	0.029 (p=0.50)
2010	26.10	1.48	46.55	1.71	2.84	0.026 (p=0.64)
2011	28.36	1.35	48.59	1.78	3.01	0.032 (p=0.38)
2012	25.87	1.47	46.56	1.73	2.98	0.036 (p=0.26)
2013	24.81	1.43	44.96	1.74	2.77	0.041 (p=0.12)
2014	26.14	1.44	46.71	1.74	2.98	0.039 (p=0.16)
2015	28.81	1.32	49.19	1.74	2.63	0.038 (p=0.19)
2016	26.35	1.35	46.86	1.75	2.95	0.035 (p=0.29)
2017	24.48	1.55	46.91	1.75	3.23	0.034 (p=0.29)

S4 Pretreatment of Flow Zero Value

For the gravity model (equation 2 in the main text), $F_{i,j}$ is the trade flow from country i to country j; m_i and m_j is the combined size of their economies; $r_{i,j}$ is the trade resistance need to be quantified. It is generally believed that the model cannot describe zero flow because the gravity is universal [1]; because even if the size of two countries is very small and the geographical distance or trade resistance is very large, as long as the volume $m_i m_j$ is not equal to zero and the resistance $r_{i,j}$ are not infinite, the trade flow between them may be very small, but not zero.

$$\begin{aligned} F_{i,j} &\simeq \frac{(m_i m_j)^\alpha}{r_{i,j}} - \varepsilon_{i,j} \\ &= exp(\alpha \ln(m_i m_j) - \ln r_{ij}) - \varepsilon_{i,j} \end{aligned} \tag{4}$$

However, the situation of zero-value flow is very common in the empirical data, around 50% in the global trade network [2], and it creates an additional problem for the log linear form of the gravity equation (including the traditional and structural gravity model in trade studies). In the early studies, some scholars often deal with the zeroes trade observation by truncation method, such as

deleting them completely or substitute by small positive constant [3, 4]. It's obviously not rigorous enough [1]. In reality, the zero-value trade flow is generally considered to be not observable or due to measurement errors from rounding. So stochastic versions of equation are used in empirical studies [5, 2]. Here we can add an error term $\varepsilon_{i,j}$, and assume that the error function is positive and obeys lognormal distribution [5], as $\ln \varepsilon_{i,j} \sim N(\mu, \sigma^2)$ in equation 4.

$$\begin{aligned} E(\varepsilon_{i,j}) &= e^{\mu^2+\sigma^2/2} \\ Var(\varepsilon_{i,j}) &= (e^{\sigma^2}-1)e^{2\mu^2+\sigma^2}. \end{aligned}$$

For clarity, we assume $X = \varepsilon_{i,j}$, and $Y = F_{i,j} = \frac{(m_i m_j)^\alpha}{r_{i,j}} - X$. The probability density function of the random variable X is,

$$f_X(x) = \begin{cases} \frac{1}{\sqrt{2\pi}\sigma x} exp[-\frac{1}{2\sigma^2}(\ln x - \mu)^2] & x > 0 \\ 0 & x \leq 0 \end{cases}$$

The probability density function of Y is calculated as follows:

$$\begin{aligned} & F_Y(y) = P(Y \leq y) = P(\frac{(m_i m_j)^\alpha}{r_{i,j}} - X \leq y) = P(X \geq \frac{(m_i m_j)^\alpha}{r_{i,j}} - y) \\ & = 1 - P(X \leq \frac{(m_i m_j)^\alpha}{r_{i,j}} - y) = 1 - F_X(\frac{(m_i m_j)^\alpha}{r_{i,j}} - y) \\ & \Rightarrow f_Y(y) = F_Y'(y) = -f_X(\frac{(m_i m_j)^\alpha}{r_{i,j}} - y) \times (-1) = f_X(\frac{(m_i m_j)^\alpha}{r_{i,j}} - y) \end{aligned}$$

If we assume that trade resistance is bilateral, then we can simply deduce $r_{i,j}$ for each pair of countries by the least square method with,

$$\begin{aligned} & min(\phi = (F_{i,j} + \varepsilon_{i,j} - \frac{(m_i m_j)^\alpha}{r_{i,j}})^2 + (F_{j,i} + \varepsilon_{j,i} - \frac{(m_i m_j)^\alpha}{r_{i,j}})^2 \\ & \frac{\partial \phi}{\partial r_{i,j}} = 0 \Rightarrow r_{i,j}^* = \frac{2(m_i m_j)^\alpha}{F_{i,j} + F_{j,i} + \varepsilon_{i,j} + \varepsilon_{j,i}} \end{aligned}$$

Different kind of Pseudo Maximum Likelihood (PML) methods are proved to be effective to deal with the zero-valued trade flow and the logarithm transformation [6, 7, 5]. The method in this paper is not exactly the same as the gravity model, and the main different is that we replace the geographical distance with $r_{i,j}$ which needs to be quantified. So we use the idea of PML, but improve the likelihood function here. Then, we maximize the probability $Y = F_{i,j}$, with the defined likelihood function as,

$$
\begin{aligned}
L &= \prod_{i,j} f_Y(F_{i,j}|\mu,\sigma) = \prod_{i,j} f_X(\frac{(m_i m_j)^\alpha}{r_{i,j}} - F_{i,j}|\mu,\sigma) \\
&= \prod_{i,j} f_X(\frac{F_{i,j}+F_{j,i}+\varepsilon_{i,j}+\varepsilon_{j,i}}{2} - F_{i,j}|\mu,\sigma) \\
&\simeq \prod_{i,j} f_X(\frac{F_{j,i}-F_{i,j}}{2} + E(\varepsilon_{i,j})|\mu,\sigma)
\end{aligned}
$$

With the method of maximum likelihood estimation, we can optimize the parameters μ and σ to get the $max_{\mu,\sigma}(L)$, which make $Y = F_{i,j}$ the most likely to occur in reality.

The optimized parameters are listed in Table S3, and Figure S2 shows the distribution of random error $\varepsilon_{i,j}$ during 2007-2017. It can be seen that the mean value of random variables is basically around 1-2, and the variance is relatively small, which conforms to the basic assumption of statistical error in trade flows.

	μ	σ	$E(\varepsilon_{ij})$
2007	0.00694	0.00050	1.00697
2008	0.00228	0.00020	1.00229
2009	0.02364	0.00047	1.02392
2010	0.56409	0.00027	1.75785
2011	0.00339	0.00024	1.00340
2012	0.01529	0.00072	1.01540
2013	0.81314	0.00018	2.25498
2014	0.05607	0.00061	1.05767
2015	0.40263	0.00017	1.49575
2016	0.31945	0.00028	1.37637
2017	0.02362	0.00047	1.02390

Table S3: Optimized Parameters

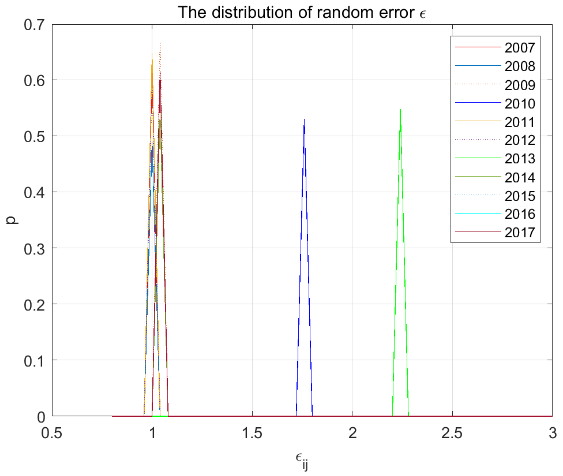

Fig. S2: The random error obeys lognormal distribution during 2007-2017.

References

1. O. I. K. Fatima Olanike Kareem, *Macro Management & Public Policies* **2019**, *01*, 01 36.
2. E. Helpman, M. Melitz, Y. Rubinstein, *The quarterly journal of economics* **2008**, *123*, 2 441.
3. R. Flowerdew, M. Aitkin, *Journal of regional science* **1982**, *22*, 2 191.
4. M. Burger, F. Van Oort, G.-J. Linders, *Spatial Economic Analysis* **2009**, *4*, 2 167.
5. J. M. C. S. Silva, S. Tenreyro, *Review of Economics Statistics* **2006**, *88*, 4 641.
6. W. G. Manning, J. Mullahy, *Journal of health economics* **2001**, *20*, 4 461.
7. I. Martínez-Zarzoso, *Applied Economics* **2011**, *45*, 3 311.
